# Drug Discovery Using Chemical Systems Biology: Weak Inhibition of Multiple Kinases May Contribute to the Anti-Cancer Effect of Nelfinavir

**DOI:** 10.1371/journal.pcbi.1002037

**Published:** 2011-04-28

**Authors:** Li Xie, Thomas Evangelidis, Lei Xie, Philip E. Bourne

**Affiliations:** 1 Skaggs School of Pharmacy and Pharmaceutical Sciences, University of California, San Diego, La Jolla, California, United States of America; 2 School of Pharmacy and Pharmaceutical Sciences, University of Manchester, Manchester, United Kingdom; 3 Department of Computer Science, Hunter College, the City University of New York, New York, United States of America; National Cancer Institute, United States of America and Tel Aviv University, Israel

## Abstract

Nelfinavir is a potent HIV-protease inhibitor with pleiotropic effects in cancer cells. Experimental studies connect its anti-cancer effects to the suppression of the Akt signaling pathway, but the actual molecular targets remain unknown. Using a structural proteome-wide off-target pipeline, which integrates molecular dynamics simulation and MM/GBSA free energy calculations with ligand binding site comparison and biological network analysis, we identified putative human off-targets of Nelfinavir and analyzed the impact on the associated biological processes. Our results suggest that Nelfinavir is able to inhibit multiple members of the protein kinase-like superfamily, which are involved in the regulation of cellular processes vital for carcinogenesis and metastasis. The computational predictions are supported by kinase activity assays and are consistent with existing experimental and clinical evidence. This finding provides a molecular basis to explain the broad-spectrum anti-cancer effect of Nelfinavir and presents opportunities to optimize the drug as a targeted polypharmacology agent.

## Author Summary

The traditional approach to drug discovery of “one drug – one target – one disease” is insufficient, especially for complex diseases, like cancer. This inadequacy is partially addressed by accepting the notion of polypharmacology – one drug is likely to bind to multiple targets with varying affinity. However, to identify multiple targets for a drug is a complex and challenging task. We have developed a structural proteome-wide off-target determination pipeline by integrating computational methods for high-throughput ligand binding site comparison and binding free energy calculations to predict potential off-targets for known drugs. Here this method is applied to identify human off-targets for Nelfinavir, an antiretroviral drug with anti-cancer behavior. We propose inhibition by Nelfinavir of multiple protein kinase targets. We suggest that broad-spectrum low affinity binding by a drug or drugs to multiple targets may lead to a collective effect important in treating complex diseases such as cancer. The challenge is to understand enough about such processes so as to control them.

## Introduction

Tremendous effort has been directed at rational drug design where one strives to understand, and subsequently optimize, how a small molecule interacts with a single protein target and impacts a disease state. However, such approaches are less fruitful in discovering effective and safe therapeutics to treat complex diseases such as cancer. It is suggested that the inhibition or activation of a single specific target may fail owing to the inherent robustness of the underlying biological networks causing the disease state [1], [2], [3], [4], [5], [6]. The goal then is to perturb multiple relevant targets. Perturbation may be achievable through the use of drug cocktails, or possibly through a single drug that has the appropriate polypharmacological effect [1], [2], [4], [6], [7], [8], [9], [10], [11]. To rationally design such a drug is a very complex problem that begins by identifying the targets to which that drug binds. Here we address a much simpler problem, that is, to take a drug that is already believed to show this effect and attempt to explain why it might be so. Nevertheless, we must still begin by identifying the multiple targets to which it binds. To this end, we have developed an off-target pipeline to identify protein-drug interaction profiles on a structural proteome-wide scale. The off-target pipeline integrates our previous chemical systems biology approach [12], [13], [14] with algorithms that accurately estimate binding affinity. We then use the target list predicted from the off-target pipeline to suggest physiological outcomes from the associated biological networks and determine how well these outcomes map to what is observed clinically.

The extension to our previous approach presented here is to better estimate the binding affinity in forming a protein-ligand complex, as both experimental and theoretical studies suggest that even weak binding to multiple targets may have profound impact on the overall biological system [15], [16], [17]. Available computational tools that quantitatively study protein-ligand interactions are based predominantly on protein-ligand docking and free energy calculations for the protein-ligand complex [18], [19]. A formidable task then is to include protein flexibility into the binding affinity calculation since errors in scoring mainly result from the use of rigid protein conformations [20]. The modeling of protein flexibility requires computationally intensive molecular dynamic (MD) simulations. However, it is impractical to apply MD simulation to the whole structural proteome. Our approach pre-filters the structural proteome to find the most likely cases to apply MD. Specifically, we undertake a human structural proteome-wide ligand binding site comparison using previously developed algorithms [21], [22], [23] and add intensive binding free energy calculations, based on protein-ligand docking, MD simulation and MM/GBSA free energy calculations.

We apply this strategy to explore the molecular mechanism for the observed anti-cancer effect of Nelfinavir, a human immunodeficiency virus (HIV) protease inhibitor. Recently, Nelfinavir has been repurposed for cancer treatment [24], [25], [26]. However, its molecular targets remain unknown. The majority of published data indicates that the drug suppresses the Akt signaling pathway [27]. In human, the Akt family includes the serine/threonine protein kinases Akt1, Akt2 and Akt3. These proteins are involved in cell survival, protein synthesis and glucose metabolism and are considered markers for many types of cancer [28], [29], [30]. Akt3 is also known to be stimulated by platelet-derived growth factor (PDGF), insulin and insulin-like growth factor 1 (IGF1) [31]. Thus inhibition of the Akt pathway may also cause insulin resistance and diabetes, a phenomenon observed as a side effect of treatment by HIV protease inhibitors. Currently, there is no experimental evidence to suggest that Nelfinavir binds directly to members of the Akt family, rather it has been suggested that the drug acts upstream of the Akt signaling pathway [32].

Using our structural proteome-wide off-target pipeline, we find that multiple members of the protein kinase-like superfamily as off-targets of Nelfinavir. Most of these protein kinases are found upstream of the Akt, MAPK, JNK, NF-κB, mTOR and focal adhesion pathways. We hypothesize that this weak but broad spectrum protein kinase inhibition by Nelfinavir contributes to the therapeutic effect against different types of cancer. Our hypothesis is supported by kinase activity assays and consistent with other existing experimental and clinical observations. This suggests that the next challenges are specifically to optimize Nelfinavir as a targeted polypharmacology agent, and more generally, to determine whether our computational protocol can be applied to other systems.

## Results

### Putative off-targets of Nelfinavir

**Figure 1:**
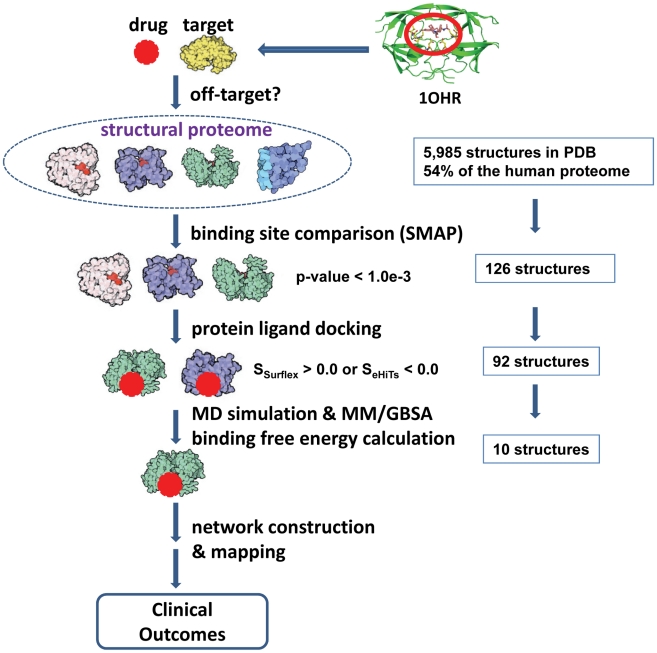
The structural proteome-wide off-target pipeline integrating ligand binding site characterization and comparison, protein-ligand docking, MD simulation and MM/GBSA energy calculations, and biological network analysis.

**Figure 2:**
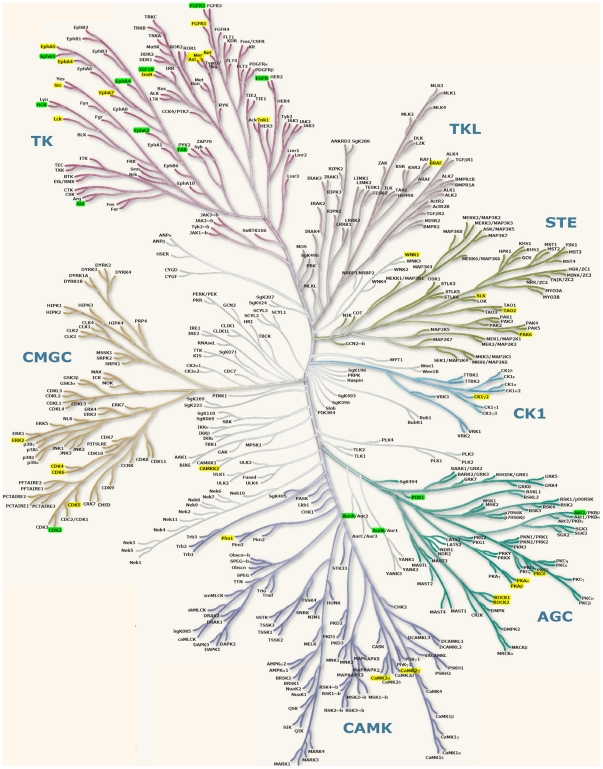
Distribution of predicted off-targets on the human kinome tree. Green represents off-targets with an SMAP p-value less than 1e-4. Yellow represents off-targets with an SMAP p-value less that than 1e-3 and greater than 1e-4.

The steps in our off-target pipeline are shown in Figure 1. In the first step, the Nelfinavir binding pocket in the HIV protease dimer structure (PDB Id: 1OHR) was used to search against 5,985 PDB structures of human proteins or homologs of human proteins using the SMAP software (see Supporting Information (SI) and methods for details), which is based on a sensitive and robust ligand binding site comparison algorithm [21], [22], [23]. Hits are considered significant if the SMAP p-value <1.0e-3. In step 2, the binding poses and affinities of Nelfinavir to these putative off-targets are estimated using two docking methods, Surflex [33] and eHiTs [34], starting from the superimposed binding sites. If the docking score indicates severe structural clashes between Nelfinavir and the predicted binding pocket, the protein is removed from the off-target list. After filtering by SMAP and the two docking programs, 92 putative off-targets remained for further analysis (SI, Table S1). Among them, the top 7 ranked off-targets belong to the aspartyl protease family that is the fusion form of the primary target HIV protease dimer. The remaining 85 proteins belong to different global folds from the primary target. These off-targets are dominated by protein kinases (PKs) (51 off-targets) and other ATP or nucleotide binding proteins (17 off-targets). The distribution of the 51 protein kinases on the human kinome tree [35] is shown in Figure 2. Even though these protein kinases have a broad distribution among the different protein kinase families, the majority of predicted off-targets belong to the tyrosine kinase, cAMP-dependent, cGMP-dependent and protein kinase C families. This distribution is more pronounced with a stringent SMAP p-value smaller than 1.0e-4 (green in Figure 2). The 12 top-ranked PKs with p-value smaller than 1.0e-4 were subject to detailed protein-Nelfinavir docking and 10 of them were further investigated through computational intensive molecular dynamic simulations and MM/GBSA binding free energy calculations.

### Predicted Nelfinavir binding to protein kinases determined by protein-ligand docking

**Figure 3:**
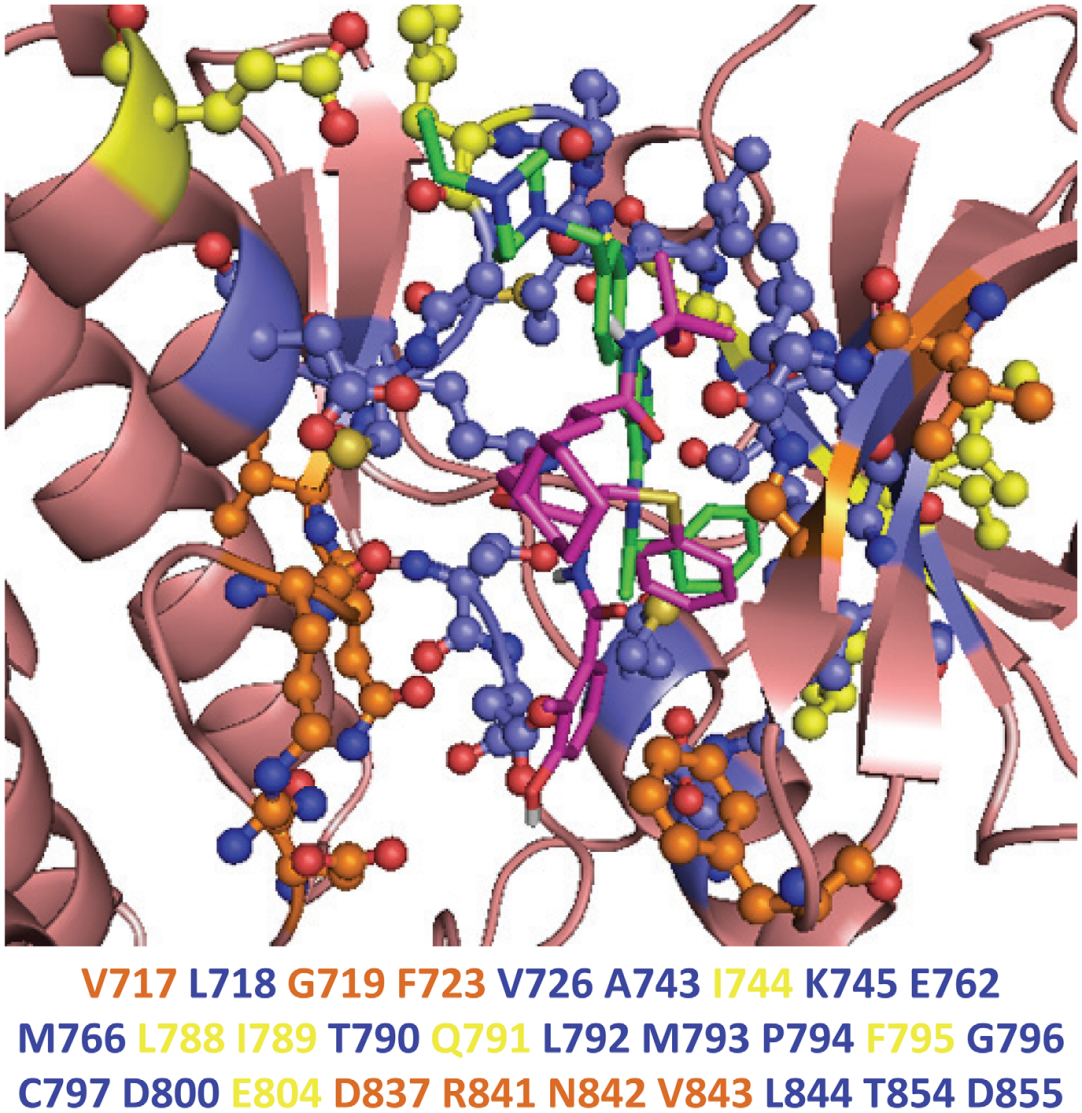
Overlap between EGFR ATP binding sites and Nelfinavir binding sites predicted by SMAP. The wheat cartoon represents the backbone structure of EGFR (PDB id: 2J6M). Green sticks represent the co-crystal ligand of EGFR (PDB ligand id: AEE). Magenta sticks represent superimposed Nelfinavir on EGFR according to SMAP alignment. Yellow sticks and balls represent the AEE binding site of EGFR. Orange sticks and balls represent the predicted Nelfinavir binding site for EGFR. Blue sticks and balls represent the overlap between the AEE binding site and the predicted Nelfinavir binding site for EGFR. The amino acid residues involved in the binding site are listed below the structure, colored accordingly.

The SMAP alignments between the PKs and the Nelfinavir binding sites reveal that ATP and its competitive inhibitors bind in the vicinity of the predicted binding sites. An example is shown in Figure 3 for the case of epidermal growth factor receptor (EGFR) protein kinase domain (PDB id: 2J6M). The superimposed Nelfinavir is accommodated in the protein kinase inhibitor binding pocket and overlaps with the co-crystallized EGFR inhibitor (PDB ligand id: AEE). If amino acid residues with atomic distances less than 5.0 Å to the inhibitors are considered as the binding site, approximately 73% (16/22) of the known AEE binding site residues are included in the predicated binding site of Nelfinavir to EGFR.

Binding poses and affinities of Nelfinavir to the identified PK binding sites are firstly estimated using the docking software eHiTs [34] and compared to the binding affinities of co-crystallized inhibitors in those PKs. The binding pose of Nelfinavir is optimized from its superimposed conformation obtained from the SMAP output rather than by an *ab initio* global conformational search. Systematic errors in the scoring function are cancelled out by using a normalized docking score (NDS) [13]. A large negative value for the NDS indicates a higher likelihood of binding. The predicated binding affinity of Nelfinavir is comparable to that of co-crystallized inhibitors for several classes of PKs, notably EGFR (SI Table S2). The NDS for EPHA2 is 1.328, which implies that the docking score of Nelfinavir to EPHA2 is higher than for randomly selected molecules. This protein was removed from further calculations.

### Ensemble average binding free energy estimation using MD simulation

In order to get more accurate estimates for the binding affinities, MM/GBSA calculations were performed on 10 PK hits filtered by the SMAP binding site similarity search and ligand docking scores. Since in reality binding is dynamic, the structure will adopt different conformations during binding, and this should be anticipated. Hence one should generate a statistically sufficient ensemble from molecular-dynamics trajectories and compare the resulting ensemble averages to obtain a more reliable binding free energy value. Recent studies on MM/GBSA binding free energy calculations show that a nanosecond scale MD simulation is sufficient to perform a meaningful MM/GBSA calculation [36], [37], [38]. Here, the binding free energies averaged over 200 snapshots from the last 2 ns trajectory of an 8 ns MD simulation are listed in Table 1 for the complex structures of protein kinases bound with Nelfinavir and co-crystallized ligands. To estimate the stability of the MD simulations, structural root-mean-square-deviations (RMSDs) for receptor backbone atoms and ligand non-hydrogen atoms are examined as a function of time (Supporting Information Figure S1). The RMSD is calculated based on superimposed structures fitting to the first frame of the 8 ns MD simulation using the coordinates of the receptor backbone atoms. Thus, RMSD values for the ligands reflect both internal and rigid body movements relative to the protein. In all cases, RMSDs for the receptor backbone atoms are well below 3 Å for the last 2 ns simulation, indicating robust simulations and reasonable samplings for the MM/GBSA binding free energy calculation. The conformational fluctuation of Nelfinavir bound to EPHB4 and FGFR are higher than in other targets. Structural analysis of their trajectories shows that Nelfinavir moves out of the binding pockets of EPHB4 and FGFR during simulation, which indicates EPHB4 and FGFR may not be good candidates for Nelfinavir interaction.

**Table 1:** Calculated binding free energies from MM/GBSA calculations for Nelfinavir and co-crystallized inhibitors for predicted off-targets.

		Ensemble average calculation(kcal/mol)		
Target	Ligand	Δ*H*_binding_	Δ*TS*_binding_	Δ*G*_binding_
ARK	HPM	-41.50	-26.70	-14.80
ARK	1UN	-41.62	-20.36	-21.26
ABL	P16	-30.14	-14.09	-16.05
ABL	1UN	-31.22	-20.41	-10.81
AKT2	I5S	-35.67	-24.38	-19.25
AKT2	1UN	-43.64	-35.67	-18.53
CDK2	1CD	-52.68	-10.10	-42.58
CDK2	1UN	-49.54	-21.67	-27.87
EGFR	AEE	-34.73	-14.16	-20.57
EGFR	1UN	-33.72	-18.12	-15.60
EPHB4	7X4	-39.61	-17.64	-21.96
EPHB4	1UN	-21.40	-19.86	-1.54
FAK	BI9	-50.02	-18.82	-31.20
FAK	1UN	-44.73	-16.20	-28.53
FGFR	SU1	-35.88	-18.44	-17.44
FGFR	1UN	-26.86	-21.24	-5.62
IGF-1R	BMI	-38.26	-17.28	-20.98
IGF-1R	1UN	-31.43	-21.37	-10.05
PDK1	BI1	-35.39	-13.65	-21.74
PDK1	1UN	-30.32	-17.76	-12.56

Ensemble averaged binding free energies calculated for 200 snapshots extracted from the last 2 ns of the MD simulation.

Here, the MM/GBSA binding free energy calculation includes gas-phase energies, solvation free energies and entropy contributions. As shown in Table 1, if only gas-phase energies and solvation free energies, i.e., total binding enthalpy, are taken into account, Nelfinavir shows comparable binding affinity to the co-crystallized ligands. However, when considering the loss of entropy during binding, Nelfinavir becomes less favorable than the co-crystallized inhibitors due to its larger size and flexibility. For example, when AEE enters the binding pocket of EGFR, the entropy change for the whole system is 14.16 kcal/mol. However, the binding of Nelfinavir to EGFR causes an 18.12 kcal/mol entropy losses for the whole system. Thus, even though the entropy contribution is smaller than the enthalpy contribution, the binding free energy difference between Nelfinavir and AEE comes predominantly from the entropy change and this part of the free energy cannot be omitted in providing a reliable estimate of binding affinity.

Ligand binding pose and atomic interactions between ligand and protein kinases are also important factors when measuring ligand binding. The predicted binding pose of Nelfinavir significantly overlaps with the known inhibitors of EGFR, IGF-1R, FAK, Akt2, CDK2, ARK and PDK1 (SI, Figure S2 and S3). The structure of Nelfinavir can be fragmented into five moieties: the 2-methyl-3-hydroxy-benzamide portion A, the S-phenyl group B, the tert-butyl carboxamide moiety C, the lipophilic dodecahydroisoquinoline ring D and the central hydroxyl group E (SI, Figure S4). The benzamide ring A in the predicted conformations superimposes well onto the aromatic groups of the co-crystallized inhibitors for these protein kinases, and plays a critical role in molecular recognition [39]. For other predicted protein kinases, the binding pose of Nelfinavir still partially overlaps with their respective co-crystallized inhibitors and occupies the ATP-binding pockets.

**Figure 4:**
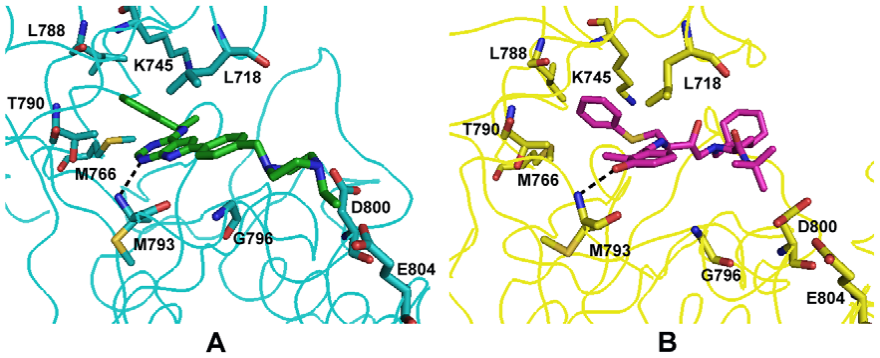
Comparative Interactions between EGFR and Inhibitor (PDB ligand id AEE; A) and EGFR and Nelfinavir (PDB ligand id 1UN; B). A: Cyan ribbon represents the backbone structure of EGFR bound to AEE (green). Cyan sticks represent the residues in contact with AEE. Black dash line represents hydrogen bonding interactions between AEE and EGFR. The distance between N and N is 3.22 Å. B: Yellow ribbon represents the backbone structure of EGFR bound to Nelfinavir. Yellow sticks represent the residues in contact with Nelfinavir. Black dash line represents hydrogen bonding interactions between Nelfinavir and EGFR. The distance between N and O is 3.16 Å.

Most of the hydrogen-bond interactions and hydrophobic interactions between protein kinases and co-crystallized inhibitors could be found between Nelfinavir and the respective protein kinases. As shown in Figure 4, the hydrogen bond between the pyrrolopyrimidine core of AEE and the main chain amide of Met793 on EGFR is maintained between benzamide hydroxy O38 of Nelfinavir and the same atom on EGFR. This hydrogen bond interaction is critical for protein-ligand binding in EGFR. Missing this hydrogen bond will cause ∼3,700-fold loss of inhibitor potency in EGFR [40]. Residues that form hydrophobic interactions with AEE are also close to Nelfinavir and provide appropriate hydrophobic interactions as shown in Figure 4. These conserved hydrogen bond interactions and hydrophobic interactions support the binding of Nelfinavir to EGFR. Similar conserved hydrogen bond interactions and hydrophobic interactions are observed for other protein kinases, excluding FGFR, EPHB4 and Abl, that is, where Nelfinavir partially overlaps with the co-crystallized ligands. The binding free energies for Nelfinavir to FGFR and EPHB4 also indicate that the binding affinities of Nelfinavir to these two proteins are weaker than the other eight protein kinases.

In summary, MM/GBSA binding free energy, ligand binding pose, conserved hydrogen bond interactions and hydrophobic interactions supports the direct interaction of Nelfinavir with EGFR, which has been shown as a possible Nelfinavir target based on ligand binding site similarity and from experimental studies by others [25]. For FGFR, EPHB4 and Abl, the results from MD simulation and MM/GBSA free energy calculations indicate that Nelfinavir is unlikely to bind to these three targets. For other targets, IGF-1R, FAK, Akt2, CDK2, ARK and PDK1, the calculated binding free energies and predicted ligand binding poses suggested the possible inhibition by Nelfinavir, even though there is no experimental support at this time.

### Protein kinase activity assay for EGFR and Akt families

**Figure 5:**
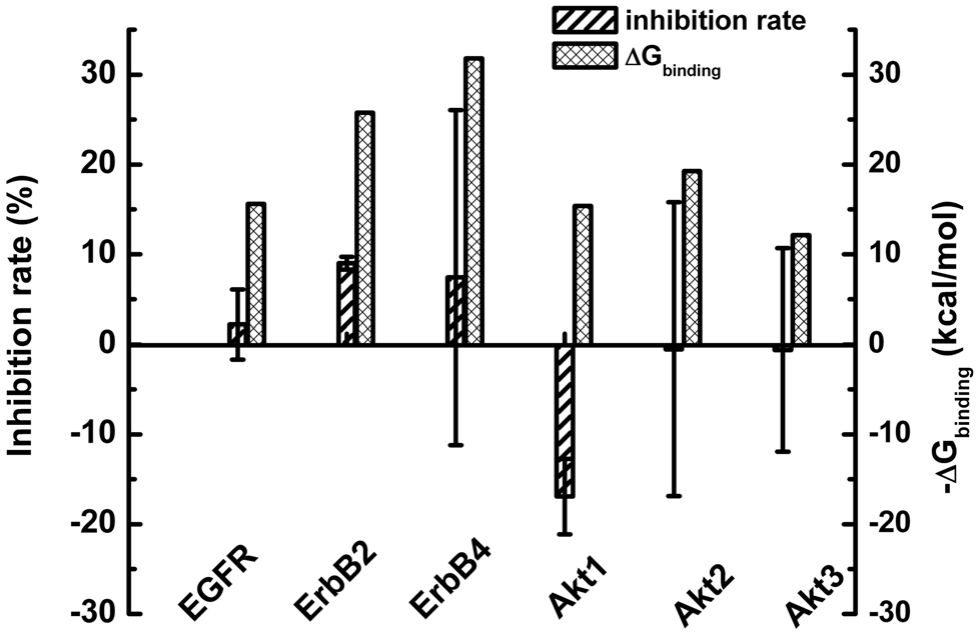
Inhibition rates and calculated MM/GBSA binding free energies of Nelfinavir binding to EGFR, ErbB2, ErbB4, Akt1, Akt2 and Akt3.

Given that computationally EGFR and Akt2 show favorable binding affinities for Nelfinavir, MD simulation and MM/GBSA binding free energy calculations were extended to other members of the EGFR (ErbB2, ErbB4) and Akt families (Akt1 and Akt3). As shown in Figure 5, the binding free energies for EGFR, ErbB2, ErbB4, Akt1, Akt2 and Akt3 are -15.60, -25.76, -31.83, -15.39, -19.25 and -12.13 kcal/mol, respectively. A HTRF® TranscreenerTM ADP Assay of 20 µM Nelfinavir was undertaken for EGFR, ErbB2, ErbB4 and Akt (Akt1, Akt2 and Akt3) in an effort to verify the predictions from the MM/GBSA calculations. Weak inhibition by Nelfinavir is detected for ErbB2 (Figure 5). The lower binding free energy of ErbB2 is consistent with its higher inhibition rate and the experimental and computational results both show inhibition of the EGFR family by Nelfinavir. Considering that a prescribed dose of Nelfinavir is 1,250 mg (2.2 mmol) (http://www.rxlist.com/viracept-drug.htm), the plasma concentration of Nelfinavir in HIV patients can reach 7-9 µM [41]. However, these concentrations only achieve a partial reduction of cancer cell proliferation and are not efficient in inducing apoptosis in cancer cells. Most cellular activity studies require concentrations of Nelfinavir greater than 20 µM [42]. At such high concentration, Nelfinavir demonstrated specific anti-cancer activity with no reports of non-specific binding. As such, it is not likely that the specific *in vivo* and *in vitro* anti-cancer activity when using a high concentration of Nelfinavir is due to its aggregation. Likewise, when the same concentration of Nelfinavir is used in our kinase assay, it is unlikely that Nelfinavir is aggregated [43]. Since the assay may not be sensitive enough to detect weak bindings, most of assay results are inconclusive. It is necessary to develop more robust assay methods for determining weak bindings.

The inhibition of EGFRs by Nelfinavir is consistent with Gills et al.’s work on exploring the effect of HIV protease inhibitors on endogenous and growth factor induced Akt activation [25]. In their study, 20 µM Nelfinavir reduced the activation of EGFR, IGF-1R and Akt signaling pathways. The decreased phosphorylation of EGFR, IGF-1R and Akt directly in response to EGF or IGF-1 indicates that Nelfinavir can compete with EGF or IGF-1 and act at the plasma membrane to inhibit growth factor receptors. However, the inhibition of Akt activation by Nelfinavir is weaker than that observed using a known PI3K inhibitor and the effect is transient, which may suggest a weaker inhibition of EGFR or IGF-1R by Nelfinavir. No obvious inhibition of Akt1 and Akt3 by 20 µM Nelfinavir is observed. Even though the ADP assay was not applied to every predicted protein kinase, the comparable computational results indicate the possibility that Nelfinavir may also inhibit other protein kinases through weak interactions.

### Comparison with other HIV protease inhibitors

Nelfinavir is the most potent inhibitor in cell proliferation and Akt activation studies [25]. To compare Nelfinavir with other protease inhibitors, MD simulation and MM/GBSA binding free energy calculations were applied to two other protease inhibitors, Saquinavir and Indinavir. Saquinavir has the most similar inhibition effect to Nelfinavir in the cell proliferation analysis involving 60 cell lines derived from nine different tumor types and Indinavir has the weakest effect on cell proliferation [25]. Autodock Vina [77] was applied to get the starting structures for Saquinavir and Indinavir when bound to EGFR, ErbB2 and ErbB4. The docking energies for Nelfinavir, Saquinavir and Indinavir are listed in SI Table S3 and show that there is no significant difference between these three inhibitors. However, the conserved hydrogen bond between Nelfinavir and EGFR cannot be found for either Saquinavir or Indinavir. The calculated MM/GBSA binding free energies for Saquinavir are -8.51, -10.12 and -9.37 kcal/mol when bound to EGFR, ErbB2 and ErbB4, respectively and -1.11, -1.68 and -2.51 kcal/mol, respectively, for Indinavir. Compared with the calculated MM/GBSA binding free energies for Nelfinavir, the less negative values for the binding free energies of Saquinavir indicate weaker binding affinities. This is consistent with the observed effect of these HIV protease inhibitors on Akt activity. The unfavorable binding of Indinavir to the EGFR families is also supported experimentally [25].

### Effect of Nelfinavir off-target binding on Akt signaling pathways

**Figure 6:**
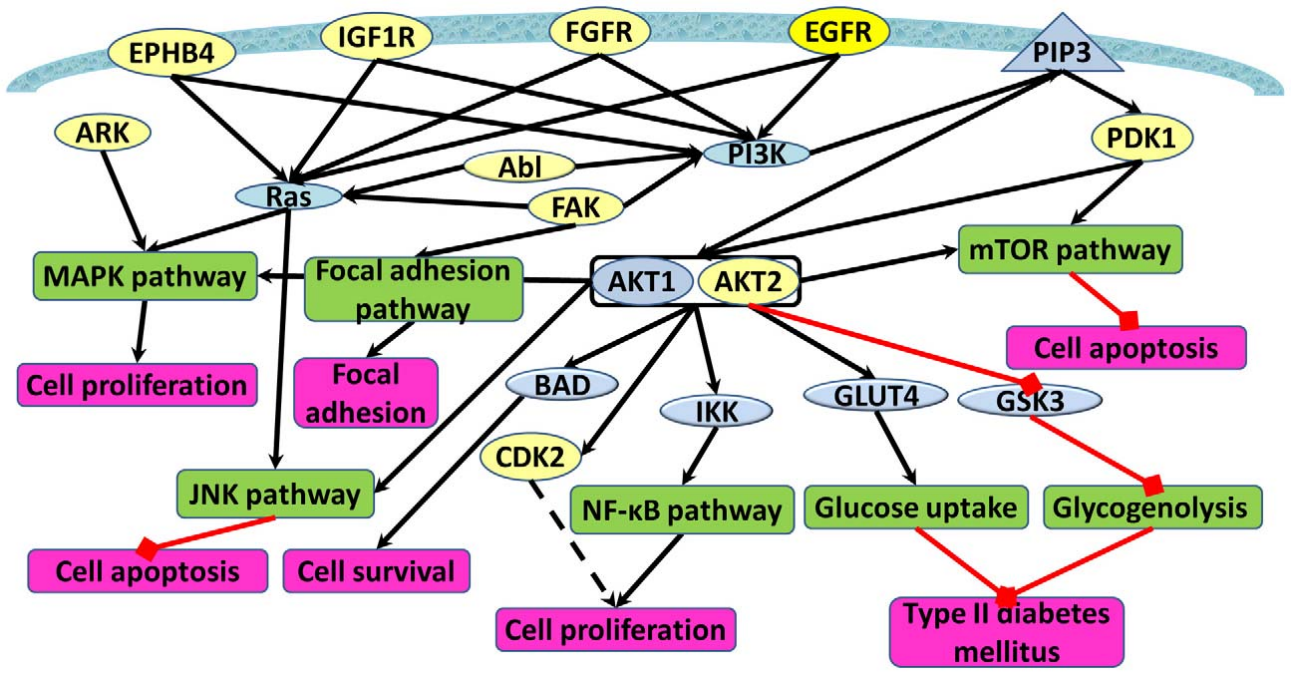
Interactions between predicted off-targets and PI3K/Akt, MAPK, JNK, NF-κB, mTOR, Glucose uptake, and Glycogenolysis pathways. Yellow circles represent predicted off-targets. Blue circle represents intermediate proteins. Green squares represent pathways. Pink squares represent cellular effects. Black lines represent activation. Red lines represent inhibition. Black dashed lines represent a dual effect (activation or inhibition).

Putting together the results from the off-target predictions, docking experiments, MD simulation, MM/GBSA free energy calculations, and kinase activity assays, it appears that Nelfinavir binds to different protein kinase (PK) off-targets through relatively weak interactions. The majority of our top ranked Nelfinavir off-targets belong to the receptor tyrosine protein kinase family, including EGFR, IGF-1R, Abl, FGFR and ephrin receptor. The PKs in this family are high affinity cell surface receptors that not only regulate normal cellular processes but also play a critical role in the development of many types of cancers. There are also other PKs identified as off targets for Nelfinavir, such as CDK2, ARK2, FAK1, Akt2 and PDK1. By examining pathways associated with each individual predicted off-target, we constructed an integrated off-target interaction network (Figure 6). To simplify the whole network, we only present the interactions between predicted off-targets and the major pathways involved in cancer development and insulin resistance. Effects of these off-targets are not limited to these pathways. Predicted off-targets, represented by yellow circles in the network, regulate PI3K, MAPK, JNK, mTOR, NF-κB and focal adhesion pathways through direct or indirect interactions with intermediate proteins connecting the pathways. Inhibition of predicted off-targets is predicted to down-regulate these pathways, and hence reduce cancer risk and increase insulin resistance.

Consider EGFR as an example to show how inhibition by Nelfinavir can result in an anti-cancer effect. Some major effects of EGFR on cellular functions come from its regulation of the PI3K/Akt pathway. As a receptor tyrosine protein kinase, EGFR can be activated by epidermal growth factor and then induce activation of Phosphoinositide 3-kinases (PI3K), resulting in the formation of a PtdIns(3,4,5)P3 molecule (PIP3 in Figure 6). Akt will then bind to PtdIns(3,4,5)P3 and be phosphorylated and activated by PDK1 and mTOR. As a consequence, the activation of Akt triggers the downstream response of the Akt pathway, such as phosphorylation of the Bcl-2-associated death promoter (BAD), activation of the NF-κB pathway and inhibition of the retinoblastoma protein (Rb). The inhibition of EGFR by Nelfinavir will reduce Akt signaling, consistent with current experimental evidence. Along with the regulation on the PI3K-Akt pathway, EGFR can also induce the activation of the MAPK and JNK pathway through interaction with Ras [44], [45]. All these activities have the potential to increase cell survival and cell proliferation and prevent cell apoptosis, as shown in Figure 6. Conversely, over-activation of EGFR and the associated down-stream pathways could result in uncontrolled cell growth and division.

Other predicted off-targets of Nelfinavir, for example, IGF-1R, Abl, FGFR, EPHB4 and FAK, have similar effects to EGFR, again by controlling activation of PI3K and Ras. According to our calculations, Nelfinavir can also bind to PDK1 and ARK. While a different mechanism than EGFR inhibition, it is hypothesized this can lead to regulation of the MAPK and mTOR pathways. PDK1 is crucial for the activation of Akt through direct phosphorylation.

CDK2 is also implicated by our off-target analysis. CDK2 is part of the downstream regulation of the PI3K/Akt pathway, and depending on cellular location, can either promote cell cycle progression or cell death [46]. The presence of active nuclear CDK2 during the transition to the G2 phase inhibits the cell cycle progression while Akt-regulated nucleo-cytoplasmic CDK2-relocation is required for cell cycle progression. The dual control of CDK2 on cell proliferation and apoptosis makes it an interesting anti-cancer target. Jiang et al. showed that Nelfinavir can inhibit CDK2 activity in melanoma cells [30] in keeping with our computational findings.

In summary, the dominant effect of Nelfinavir through off-target binding to a variety of protein kinases comes from up-stream regulation of the PI3K/Akt pathway. These protein kinases are also hypothesized to regulate other cancer pathways such as MAPK, JNK, NF-κB, mTOR and the focal adhesion pathway. Similarly, Nelfinavir is predicted to inhibit IGF-1R, which regulates the insulin/insulin-like growth factor signaling pathway, and offers one possible explanation for the observed side effects of Nelfinavir on insulin resistance and diabetes.

## Discussion

This study indicates that Nelfinavir is capable of a broad based polypharmacological effect against a number of protein kinases as targets. Determining the total number of possible targets is limited by the availability of the 3-D structures (or models) of human proteins. A second limitation might arise based on the versatility of Nelfinavir itself. The binding sites determined here map to the image of the ligand in the conformation it is found when bound to an HIV-1 protease. It might bind to a different target using a different conformation with higher affinity than observed here and these would not be found since the binding pocket itself would be different.

Given that existing experimental data indicate that the off-targets to Nelfinavir are involved in the Akt pathway, other potential strong binding off-targets upstream of the identified receptor tyrosine kinases also need to be considered. One of the most likely alternatives is the β-arrestin regulated G-protein coupled receptor signaling transduction pathway which regulates MAPKs, SRC, PI3K, and Akt, and mediates EGFR transactivation [47]. Two major non-kinase proteins involved in the kinase regulation and transactivation of the GPCR signaling pathway are the GPCR and β-arrestin. If the GPCR or β-arrestin is strongly inhibited by Nelfinavir, it is expected that the cellular functions such as GPCR internalization, translocation of smoothened to the primary cilium, and chemotaxis control, which are mediated by the β-arrestin, should be affected [47]. However, the related phenotype changes have not been reported. In addition, no significant hits (p-value <1.0e-5) are found for Nelfinavir using the Similarity Ensemble Approach (SEA), which is one of the most sensitive methods to identify GPCR related off-targets [48], [49]. The SMAP similarity between β-arrestin and HIV protease is not significant (p-value >1.0e-2). Although more analyses are required to determine if Nelfinavir binds to other proteins that indirectly regulate EGFR pathways, the data reported here at least suggest that the pleotropic effect of Nelfinavir comes from the direct inhibition of a variety of protein kinases.

A fundamental question raised by this work is whether weak binding of a drug to multiple targets can cumulatively cause strong phenotypic changes? Existing studies of biological networks have shown that the malfunction of multiple nodes more likely causes the system to fail than the removal of a single node as a result of diversity, redundancy and system control of the biological network. Multiple node failures have been called “fail-on” [50], and used to explain neurological disorders [51] and cancer [52], [53] in recent genome-wide studies. Addressing the fail-on phenomenon would require a polypharmacological effect. The therapeutic efficacy of multiple protein kinase inhibitors suggested here has already been demonstrated by less specific protein kinase inhibitors which attack tumors through multiple mechanisms and are used in more than one type of cancer therapy [54]. For example, Sunitinib is the first cancer drug simultaneously approved for two different cancer treatments, namely, renal cell carcinoma and imatinib-resistant gastrointestinal stromal tumor. A protein kinase assay against 113 different kinases shows that Sunitinib can bind to 73 additional kinases apart from its primary target [55]. In another example, moderate micomolar RAF inhibitor PLX4720 is potent in inhibiting downstream signaling and proliferation of the cell harboring BRAF, and in treating melanoma cell lines [56]. In contrast, Sorafenib that was developed as a potent nanomolar RAF inhibitor failed in the clinical trial due to its low anti-melonoma efficacy. Araujo et al. demonstrated the synergistic effect of multiple low-dose inhibition of upstream processes on the attenuation of downstream signals in the EGFR signaling pathway [57], and suggested that low-dose combination therapy may reduce drug side effects and resistances in the treatment of cancer [58]. Nelfinavir is a potential lead compound in the design of the next generation of anti-cancer drugs. As indicated by the MM/GBSA binding free energies for different protein kinases, the binding affinity of Nelfinavir is weaker than for the original inhibitors. Entropy changes during binding contribute significantly to the differences in binding affinity since Nelfinavir consists of more rotatable bonds and is more flexible than many small molecule protein kinase inhibitors. Covalent bonds could be added to the Nelfinavir structure to reduce the degree of freedom and increase the specificity and binding affinity. On the other hand, it can be hypothesized that the weak binding of Nelfinavir to multiple protein kinases helps avoid severe side effects, but still impacts the system enough to have a positive effect. That is, weak inhibition of multiple protein kinases may be just enough to return the system to a normal state, as suggest by dynamic analysis of model systems [57], [58].

There are a number of unmet computational challenges in exploiting the concept of multiple weak interactions and designing selective polypharmacology therapeutics, from target identification to lead optimization. Computational techniques that are able to identify optimal combination targets and their inhibition windows in cellular networks have been developed, but their scope is still limited [59], [60], [61]. It is well accepted that an optimal lead should balance binding potency and molecular size [62]. A highly potent lead compound usually leads to a drug candidate with high molecular weight, which is often linked to a higher risk of failure in drug development [63]. Analysis of the binding affinity of marketed drugs and natural products indicates that therapeutic efficacy is not necessarily associated with high binding affinity [63]. Moreover, drug-target interactions *in vivo* are different from those *in vitro.* An increasing body of evidence suggests that the drug-target residence time, a measurement of the lifetime of the drug-target complex, better correlates to drug efficacy than does the binding affinity [64], [65]. This suggests that lead optimization should focus on the drug-target residence time instead of binding affinity. Although methodologies have been proposed for multi-target screening based on binding affinity [66], there are simply no computational tools available for the efficient and accurate *a priori* estimation of the drug-target residence time from molecular structures.

A detailed understanding of the effect of multiple interactions on the biological network requires innovative systems biology approaches. The qualitative description of the biological network presented here is limited in its predictive power, considering the highly dynamic nature of signal transduction pathways. A mathematical modeling approach will be more powerful than the static approach as we have demonstrated recently in a study of CETP inhibitors [67]. Existing mathematical modeling methods such as ordinary differential equations, Petri nets, and pi-calculus require a large number of kinetics parameters to simulate the dynamic behavior of the biological system [68]. In practice many of these parameters may not be available. Thus the network model has to be reduced. The qualitative properties derived from off-target binding network may help to develop restrained but functional dynamic models that are suitable for parameter optimization and mathematical modeling.

In conclusion, by integrating methods from structural bioinformatics, molecular modeling and network analysis, we propose that the observed anti-cancer effects of the HIV protease inhibitor Nelfinavir derive from weak binding to multiple protein kinases that are mostly upstream of the PI3K/Akt pathway. Our computational approach, enhanced from previous work with the use of MD simulation and MM/GBSA free energy calculations, is supported by kinase activity assays and existing experimental and clinical evidence. This type of approach has the potential to be generalized as a form of rational polypharmacological drug design.

## Materials and Methods

### Overview of structural proteome-wide off-target pipeline

The structural proteome-wide off-target pipeline is outlined in Figure 1. Firstly, the Nelfinavir binding pocket in the HIV protease (PDB Id: 1OHR) was used to search against 5,985 PDB structures of human proteins or homologous of human proteins using the SMAP software [21], [22], [23]. Secondly, the binding poses and affinities of Nelfinavir to these putative off-targets are estimated using two docking methods, Surflex [33] and eHiTs [34]. If the docking score indicates severe structural clashes between Nelfinavir and the predicted binding pocket, the protein is removed from the off-target list. Finally, the remaining putative off-targets are subject to MD simulation, MM/GBSA calculation, network reconstruction, and kinase activity assay.

### Ligand binding site similarity search

5,985 PDB structures that are homologous to human proteins (sequence identify >30%, alignment coverage larger than 90%) are searched against the HIV-1 protease dimer (PDB id: 1OHR) using SMAP, which can be downloaded from http://funsite.sdsc.edu. The detailed algorithms implemented in SMAP are presented elsewhere [21], [22], [23]. In brief, proteins are represented using Cα atoms only and characterized by a geometric potential [21]. Then two proteins are aligned to identify similar local binding sites using the Sequence Order Independent Profile-Profile Alignment (SOIPPA) algorithm [22]. The statistical significance of the binding site similarity is estimated using an extreme value distribution model [23].

### Reverse docking of the human structural proteome

The binding affinity of Nelfinavir to the putative off-targets with SMAP p-value less than 1.0e-3 are estimated by two docking methods, Surflex [33] and eHiTs [34]. First, the complex structure of HIV-1 protease with Nelfinavir is superimposed onto these proteins according to the SMAP alignment. The superimposed structure of Nelfinavir is used as the starting conformation for docking. The binding pose of Nelfinavir in these statistically significant off-targets is locally optimized and scored starting from the starting conformation using Surflex 2.1 (default setting) and eHits 6.2 (the fastest setting). The docking score is normalized using the protocol described in reference [13].

### MD simulation and MM/GBSA binding free energy calculation

MM/GBSA [69], [70] was developed for free energy calculations and has been used to estimate the binding affinity for several protein or DNA systems [71], [72], [73], [74]. Here we perform ensemble average MM/GBSA binding free energy calculation on the snapshots from the MD simulation to compare binding affinity of Nelfinavir with that of the co-crystallized ligands.

### MD simulation on the complex structures of predicted protein kinase off-targets and their inhibitors including Nelfinavir

Explicit solvent molecular dynamics simulations were performed with NAMD [75] on the structures of the Nelfinavir-protein kinase complexes and co-crystallized ligand-protein kinase complexes. The starting structure for Nelfinavir in each protein kinase is the lowest energy conformation obtained through Autodock Vina [76]. These complex structures are embedded in rectangular boxes of TIP3P water [77] molecules to mimic the solvent environment. The smallest distances between the edge of the boxes and the atoms of the complex structures are adjusted to be at least 10 Å. Ions are added to neutralize these systems and satisfy the salt concentrations. The salt concentration is obtained from individual experimental condition for each protein kinase. The long distance cut-off for both van der Waals interactions and electrostatic interactions is set as 14 Å. A switching function is used to truncate the van der Waals energy smoothly at the cut-off distance. The Particle Mesh Ewald (PME) [78] method is applied to treat the long range electrostatic interactions. All covalent bonds involving hydrogen atoms are constrained by the SHAKE algorithm [79]. In order to simulate the NPT ensemble (system with a fixed pressure P, temperature T, and number of atoms N), the Langevin piston Nose-Hoover method [80], [81] in NAMD together with the periodic boundary conditions is used to maintain a constant pressure and temperature for these systems.

Systems are first minimized by a five-stage minimization protocol. Hydrogen atoms are optimized in the first stage, keeping all other atoms fixed. Then water molecules and side chain atoms are relaxed in the second and third stage, respectively. All atoms are optimized in the fourth stage, with position restraints on backbone atoms of proteins and ligands. Minimization is completed by an additional 25,000 steps, without any restraints, to remove bad contacts. All minimizations are preformed with the conjugate gradient energy minimization method [82] in NAMD.

The optimized systems are then gradually heated from 0 K to 50K, 100 K, 150 K, 200 K, 250 K, and experimental temperature (about 298 K) with position restraints on the backbone atoms. The structures are equilibrated at each temperature for 250 ps with a 1.0 fs time step. The force constant of restraints is 4.0 kcal/mol·Å^-2^. After the systems are heated to the experimental temperature, position restraints are removed in the following 120 ps simulation by gradually reducing the force constant. Subsequently 8 ns NPT MD simulations are carried out on these systems with 1.0 fs time step at the experimental temperature. 200 snapshots are extracted from the last 2 ns simulations with 10.0 ps time intervals to generate representative configurations for the MM/GBSA binding free energy calculation.

### MM/GBSA calculation

The binding free energy can be calculated through the following equation:
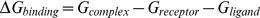
(1)where *G_complex_*, *G_receptor_*, *G_ligand_* are the free energies of the complex, receptor and ligand respectively. The free energy of each molecular on the right hand side can be considered as the sum of molecular mechanical energy in gas phase, solvation energy and entropy term, as shown in the following formula:
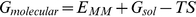
(2)


*E_MM_* is calculated by the molecular mechanics method with standard force field parm9 in AMBER9 package [83], [84]. The electrostatic contribution to the solvation free energy is determined by the Generalized Born (GB) model [85], [86], [87], [88], a widely used continuum solvent model. The “OBC” model with modified Bondi radii (mbondi2) [89], [90], [91] in AMBER9 is applied to calculate this part of energy. This model is newer than the original version of the GB model and provides a significant improvement and is recommended for both proteins and nucleic acids. The interior dielectric constant of the molecule of interest is set as 1.0 and the exterior or solvent dielectric constant is set as 78.5. The non-polar contribution to the solvation free energy is proportional to the solvent-accessible surface area [89], [92]. The surface area is calculated by the LCPO model [93] and the surface tension used to calculate the non-polar part is taken as 0.0072 kcal/mol·Å^-2^. The entropic term is the most time-intensive part of the MM/GBSA calculation but is found to be indistinguishable among different conformational states and contributes less than the other two terms in many application for estimating relative binding free energies [69], [94], [95]. The entropy change associated with ligand binding is estimated by normal mode analysis [96] in AMBER9. For each system, the MM/GBSA calculation is carried out on the 200 snapshots extracted from the last 2ns of the MD simulation.

### Protein kinase activity assay

HTRF® TranscreenerTM ADP Assays were performed on EGFR, ErbB2 and ErbB4 Akt1, Akt2 and Akt3 by GenScript (New Jersey, U.S.A). Nelfinavir Mesylate was purchased from Toronto Research Chemicals (North York, Canada). The compound is diluted to a 10 mM concentration with acetone and stored at -20°C. Inhibition of Nelfinavir at 20 µM was tested on EGFR, ErbB-2, ErbB-4 and Akt (Akt1, Akt2, Akt3).

## Supporting Information

Figure S1 Structural root mean square deviations (RMSDs) for receptor backbone atoms and ligand non-hydrogen atoms as a function of simulation time.
(DOC)

Figure S2 Comparison between binding poses of predicted Nelfinavir and co-crystallized inhibitors after MD simulation for the four receptor tyrosine protein kinases, EGFR, IGF-1R, FGFR and EPHB4.
(DOC)

Figure S3 Comparison between binding poses of predicted Nelfinavir and co-crystallized inhibitors after MD simulation for FAK, Akt2, CDK2, Abl, ARK and PDK1.
(DOC)

Figure S4 Structure of Nelfinavir.
(DOC)

Table S1 SMAP p-values, Surflex docking scores and eHiTs docking scores for putative off-targets of Nelfinavir.
(DOC)

Table S2 Comparison of normalized docking score (NDS) of Nelfinavir and co-crystallized ligands to the predicted PK off-targets.
(DOC)

Table S3 Comparison of Autodock Vina energies normalized of Nelfinavir, Saquinavir and Indinavir when bound to EGFR, ErbB2 and ErbB4.
(DOC)
