# Supplementary material for: Drug Discovery Using Chemical Systems Biology: Weak Inhibition of Multiple Kinases May Contribute to the Anti-Cancer Effect of Nelfinavir
Source: PLoS Comput Biol. 2011 Apr 28;7(4):e1002037. doi: 10.1371/journal.pcbi.1002037 (PMC3084228; doi:10.1371/journal.pcbi.1002037)
Supplement: Figure S1 — Structural root mean square deviations (RMSDs) for receptor backbone atoms and ligand non-hydrogen atoms as a function of simulation time. (DOC) [file pcbi.1002037.s001.doc]

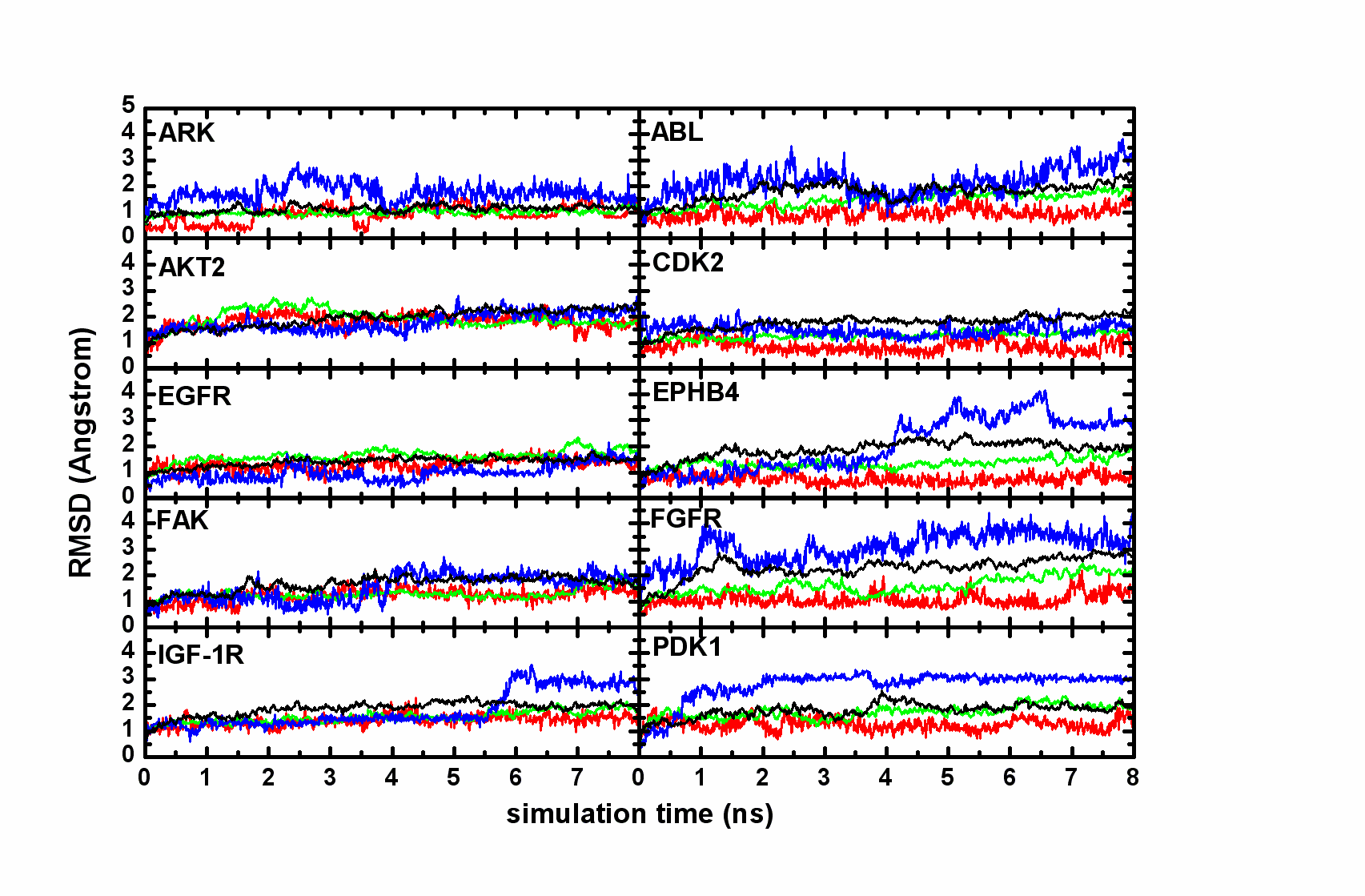


**Figure S1. Structural root mean square deviations (RMSDs) for receptor backbone atoms and ligand non-hydrogen atoms as a function of simulation time.** Red line represents RMSDs of non-hydrogen atoms for co-crystal ligands. Green line represents RMSDs of backbone atoms for receptors bound to the co-crystal ligand. Blue represents RMSDs of non-hydrogen atoms for Nelfinavir. Black line represents RMSDs of backbone atoms for receptors bound to Nelfinavir.
