# Supplementary material for: Drug Discovery Using Chemical Systems Biology: Weak Inhibition of Multiple Kinases May Contribute to the Anti-Cancer Effect of Nelfinavir
Source: PLoS Comput Biol. 2011 Apr 28;7(4):e1002037. doi: 10.1371/journal.pcbi.1002037 (PMC3084228; doi:10.1371/journal.pcbi.1002037)
Supplement: Figure S2 — Comparison between binding poses of predicted Nelfinavir and co-crystallized inhibitors after MD simulation for the four receptor tyrosine protein kinases, EGFR, IGF-1R, FGFR and EPHB4. (DOC) [file pcbi.1002037.s002.doc]

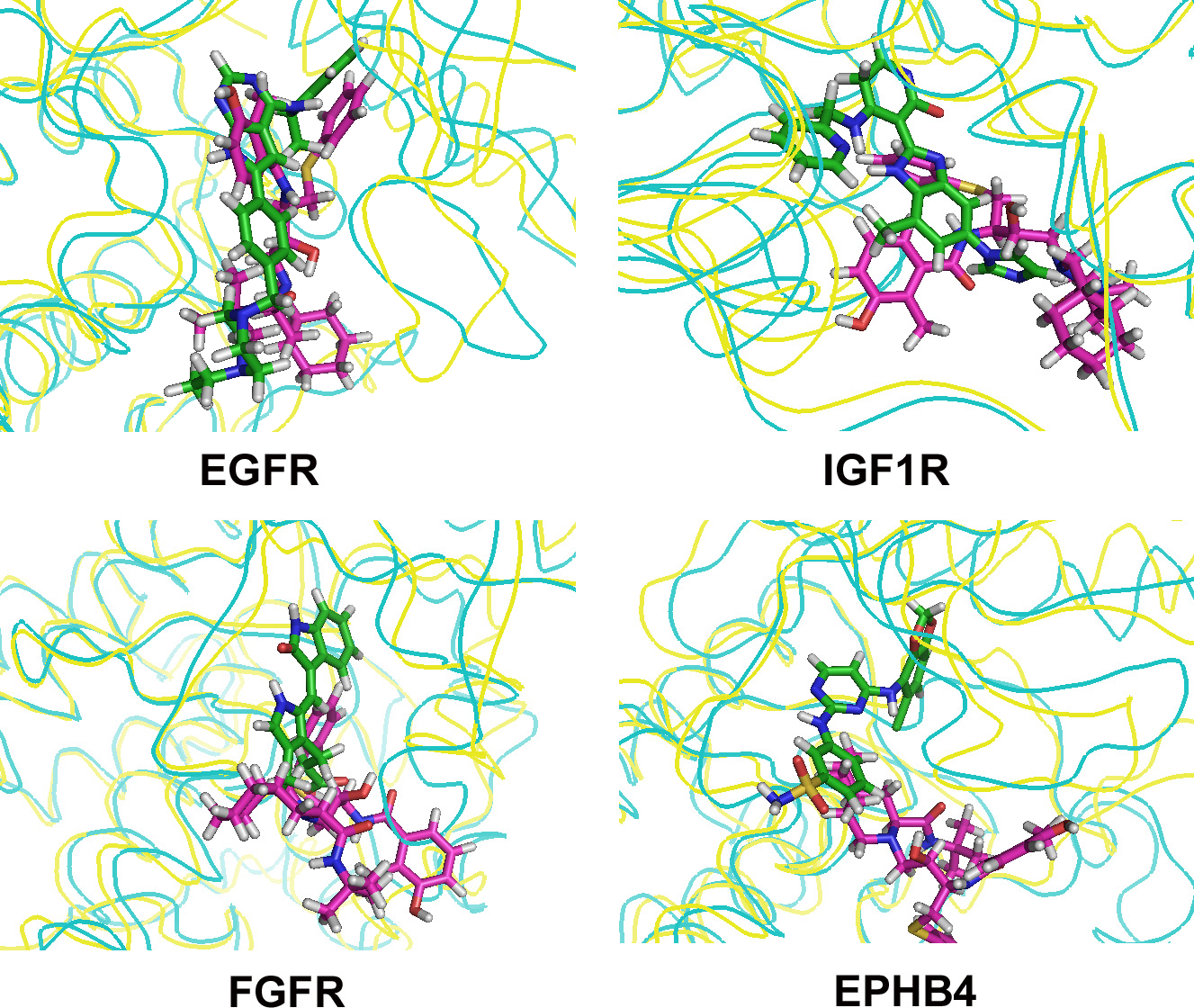


**Figure S2.** **Comparison between binding poses of predicted Nelfinavir and co-crystallized inhibitors after MD simulation for the four receptor tyrosine protein kinases, EGFR, IGF-1R, FGFR and EPHB4.** Cyan ribbon represents backbone structure of protein kinase bound with co-crystallized inhibitors. Yellow ribbon represents backbone structure of protein kinase bound with Nelfinavir. Green sticks represent structure of co-crystallized inhibitors. Magenta sticks represent structure of Nelfinavir.
