# Supplementary material for: Drug Discovery Using Chemical Systems Biology: Weak Inhibition of Multiple Kinases May Contribute to the Anti-Cancer Effect of Nelfinavir
Source: PLoS Comput Biol. 2011 Apr 28;7(4):e1002037. doi: 10.1371/journal.pcbi.1002037 (PMC3084228; doi:10.1371/journal.pcbi.1002037)
Supplement: Figure S3 — Comparison between binding poses of predicted Nelfinavir and co-crystallized inhibitors after MD simulation for FAK, Akt2, CDK2, Abl, ARK and PDK1. (DOC) [file pcbi.1002037.s003.doc]

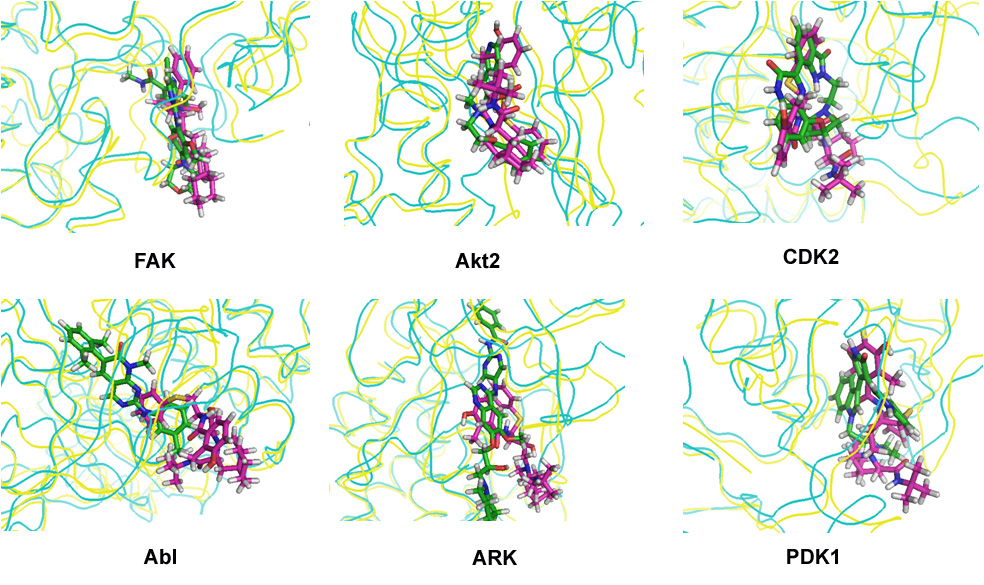


**Figure S3. Comparison between binding poses of predicted Nelfinavir and co-crystallized inhibitors after MD simulation for FAK, Akt2, CDK2, Abl, ARK and PDK1.** Cyan ribbon represents backbone structure of protein kinase bound with co-crystallized inhibitors. Yellow ribbon represents backbone structure of protein kinase bound with Nelfinavir. Green sticks represent structure of co-crystallized inhibitors. Magenta sticks represent structure of Nelfinavir.
