# Supplementary material for: Drug Discovery Using Chemical Systems Biology: Weak Inhibition of Multiple Kinases May Contribute to the Anti-Cancer Effect of Nelfinavir
Source: PLoS Comput Biol. 2011 Apr 28;7(4):e1002037. doi: 10.1371/journal.pcbi.1002037 (PMC3084228; doi:10.1371/journal.pcbi.1002037)
Supplement: Figure S4 — Structure of Nelfinavir. (DOC) [file pcbi.1002037.s004.doc]

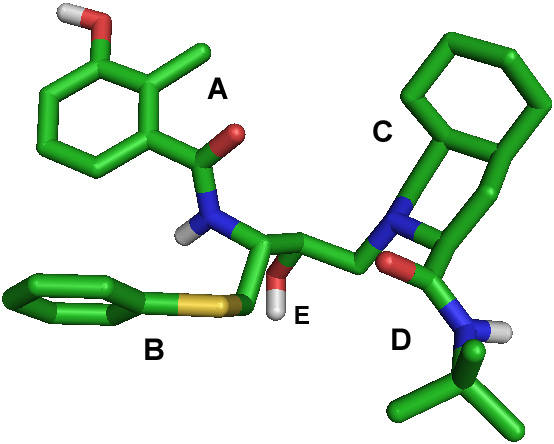


**Figure S4. Structure of Nelfinavir**. A: 2-methyl-3-hydroxy-benzamide moiety; B: S-phenyl group; C: lipophilic dodecahydroisoquinoline ring; D: tert-butyl carboxamide moiety; E: central hydroxyl group.
