# Supplementary material for: Drug Discovery Using Chemical Systems Biology: Weak Inhibition of Multiple Kinases May Contribute to the Anti-Cancer Effect of Nelfinavir
Source: PLoS Comput Biol. 2011 Apr 28;7(4):e1002037. doi: 10.1371/journal.pcbi.1002037 (PMC3084228; doi:10.1371/journal.pcbi.1002037)
Supplement: Table S1 — SMAP p-values, Surflex docking scores and eHiTs docking scores for putative off-targets of Nelfinavir. (DOC) [file pcbi.1002037.s005.doc]

**Table S1. SMAP p-values, Surflex docking scores and eHiTs docking scores for putative off-targets of Nelfinavir**

**Protein SMAP Surflex eHiTs**

**p-value Score Score**

--------- ---------- ---------- ---------

renin 1.729e-9 0.98 -5.348

uropepsin 2.165e-9 3.43 -5.536

pepsin 3A 9.524e-9 3.66 -4.870

beta-secretase 1 1.544e-8 3.55 -3.977

gastricsin 1.134e-7 1.72 -2.121

prohibitin 1.205e-6 2.96 -2.787

beta-secretase 2 2.759e-6 2.61 -3.299

insulin-like growth factor 1 receptor 5.496e-6 2.92 -5.472

tyrosine-protein kinase ABL1 6.269e-6 5.19 -5.566

glycerol kinase 1.228e-5 3.46 -3.876

cell division protein kinase 2 2.483e-5 5.62 -5.727

acetylcholine receptor protein, gamma chain 2.701e-5 1.11 -3.333

fibroblast growth factor receptor 2 3.408e-5 4.07 -5.195

epidermal growth factor receptor 3.762e-5 5.90 -7.763

aurora-related kinase 2 3.810e-5 3.55 -5.587

focal adhesion kinase 1 4.115e-5 4.41 -4.872

tyrosine-protein kinase HCK 4.343e-5 4.32 -5.611

ephrin type-A receptor 2 4.462e-5 4.68 -4.035

pyruvate kinase 5.209e-5 2.04 -2.509

SAM-dependent methyltransferase 6.511e-5 1.39 -5.565

RAC-beta serine/threonine-protein kinase (AKT2) 6.514e-5 4.86 -7.117

ephrin type-B receptor 4 7.769e-5 5.16 -4.440

3-phosphoinositide dependent protein kinase 1 8.888e-5 4.14 -5.649

benzoate-coenzyme A ligase 1.119e-4 4.19 -2.711

ADP-dependent glucokinase 1.214e-4 5.84 -4.378

ephrin type-A receptor 7 1.221e-4 1.78 -3.552

dual specificity mitogen-activated protein kinase 1 1.233e-4 3.23 -4.846

interleukin-1 receptor-associated kinase 4 1.238e-4 4.89 -4.454

NADH-quinone oxidoreductase 1.294e-4 3.90 -5.230

tyrosine-protein kinase src 1.345e-4 3.58 -6.210

beta-actin 1.412e-4 2.71 -5.446

cell division protein kinase 6 1.460e-4 3.29 -6.104

reelin 1.472e-4 2.61 -4.748

Rho-associated protein kinase 2 1.490e-4 4.24 -5.036

dihydroxyacetone kinase 1.501e-4 3.84 -9.841

phosphatidylinositol 3-kinase 1.538e-4 5.84 -5.675

acetyl-coenzyme a synthetase 1.551e-4 1.44 -3.652

cyclin-dependent protein kinase 5 1.628e-4 0.88 -2.974

transitional endoplasmic reticulum atpase 1.713e-4 1.44 -5.887

nagk protein 1.779e-4 0.66 -3.573

m-calpain 1.839e-4 1.00 -4.177

rho-associated protein kinase 1 1.953e-4 1.70 -4.361

angiopoietin-1 receptor 2.130e-4 0.34 -3.506

cyclin-dependent protein kinase 2 2.199e-4 3.70 -4.718

protein-l-isoaspartate(D-aspartate)-o-methyltransferase 2.363e-4 4.06 -5.636

cytochrome b 2.412e-4 1.36 -6.254

RNA uridylyl transferase 2.501e-4 3.23 -5.116

dihydropyrimidine dehydrogenase 2.522e-4 1.99 -4.172

G-protein coupled receptor kinase 2 2.570e-4 3.59 -4.219

pantothenate kinase 1 2.706e-4 3.24 -3.977

PSD-95 SH3-guanylate kinase domain 2.826e-4 0.30 -3.825

complement c3 beta chain 3.039e-4 1.12 -5.033

cyclin-dependent kinase 4 3.127e-4 0.33 -5.455

fgf receptor 1, kinase domain 3.129e-4 1.17 -4.106

serine/threonine kinase 6 3.253e-4 4.24 -5.529

ribosomal protein 3.254e-4 3.26 -3.908

casein kinase 1 gamma 2 3.325e-4 4.72 -6.292

cytochrome p450 2c8 3.523e-4 2.36 -6.926

activated cdc42 kinase 1 3.650e-4 4.56 -5.352

serine/threonine-protein kinase/endoribonuclease IRE1 3.692e-4 3.42 -2.533

serine/threonine-protein kinase B-RAF 3.818e-4 1.75 -2.512

pyruvate dehydrogenase kinase 3.895e-4 4.53 -5.914

tyrosine-protein kinase receptor RET 4.038e-4 3.70 -4.862

serine/threonine protein kinase TAO2 4.049e-4 3.51 -2.945

protein kinase C, theta type 4.170e-4 4.48 -5.013

DNA polymerase iii alpha subunit 4.211e-4 2.13 -3.847

tyrosine-protein kinase ERBB-4 4.258e-4 4.16 -5.799

amyloid protein-binding protein 4.316e-4 2.29 -3.493

pyrroline-5-carboxylate reductase 1 4.796e-4 3.02 -5.738

tyrosine-protein kinase mer 4.981e-4 3.50 -5.899

vascular endothelial growth factor receptor 2 5.112e-4 2.31 -6.496

tyrosine-protein kinase lck 5.200e-4 4.70 -7.084

ero1p 5.458e-4 0.33 -3.997

heat shock cognate 5.699e-4 5.99 -3.352

adenylyltransferase THIF 5.737e-4 1.81 -4.834

serine/threonine-protein kinase PIM-1 5.769e-4 4.83 -6.347

SAM-dependent methyltransferase, REBM 5.920e-4 3.93 -5.211

cAMP-dependent protein kinase 6.190e-4 5.55 -4.932

erine/threonine protein kinase TAO2 6.402e-4 2.87 -2.743

serine/threonine-protein kinase pknB 6.482e-4 2.80 -5.389

putative asparaginyl hydroxylase 6.910e-4 3.72 -2.638

mitogen-activated protein kinase 8 7.322e-4 2.89 -7.800

vitamin d binding protein 7.497e-4 1.14 -1.200

3-isopropylmalate dehydrogenase 7.694e-4 1.92 -1.503

serine/threonine-protein kinase 24 7.790e-4 4.17 -7.269

ephrin type-A receptor 5 8.027e-4 5.41 -5.049

TGF-A superfamily receptor type I 8.442e-4 4.86 -5.119

insulin receptor subunit beta 8.631e-4 2.63 -2.617

serine/threonine-protein kinase wnk1 9.213e-4 1.11 -1.842

IGF-1 receptor kinase 9.570e-4 3.26 -4.604

cyclin-dependent protein kinase pho85 9.577e-4 1.53 -3.421

kinesin heavy chain-like protein 9.983e-4 2.72 -3.951
