# Supplementary material for: Drug Discovery Using Chemical Systems Biology: Weak Inhibition of Multiple Kinases May Contribute to the Anti-Cancer Effect of Nelfinavir
Source: PLoS Comput Biol. 2011 Apr 28;7(4):e1002037. doi: 10.1371/journal.pcbi.1002037 (PMC3084228; doi:10.1371/journal.pcbi.1002037)
Supplement: Table S2 — Comparison of normalized docking score (NDS) of Nelfinavir and co-crystallized ligands to the predicted PK off-targets. (DOC) [file pcbi.1002037.s006.doc]

**Table S2. Comparison of normalized docking score (NDS) of Nelfinavir and co-crystallized ligands to the predicted PK** off-targets.

| **Protein** | **Co-crystal ligand** | **Co-crystal ligand NDS** | **Nelfinavir NDS** |
| --- | --- | --- | --- |
| insulin-like growth factor 1 receptor (IGF-1R) | BMI | -1.513 | -0.579 |
| tyrosine-protein kinase (ABL1) | 4ST | -0.787 | -0.107 |
| cell division protein kinase 2 (CDK2) | 1CD | -2.131 | -0.989 |
| fibroblast growth factor receptor 2 (FGFR) | SU1 | -1.376 | -1.170 |
| epidermal growth factor receptor (EGFR) | AEE | -2.221 | -2.116 |
| aurora-related kinase 2 (ARK) | HPM | -2.222 | -0.171 |
| focal adhesion kinase 1 (FAK) | BI9 | -1.683 | +0.397 |
| tyrosine-protein kinase HCK (HCK) | L1G | -1.451 | -0.228 |
| ephrin type-A receptor 2 (EPHA2) | CC3 | -1.161 | +1.328 |
| RAC-β serine/threonine-protein kinase (AKT2) | G96 | -1.884 | -1.534 |
| ephrin type-B receptor 4 (EPHB4) | 7X2 | -1.998 | +0.203 |
| 3-phosphoinositide dependent protein kinase 1 (PDK1) | BI1 | -1.432 | -0.104 |
