# Supplementary material for: Drug Discovery Using Chemical Systems Biology: Weak Inhibition of Multiple Kinases May Contribute to the Anti-Cancer Effect of Nelfinavir
Source: PLoS Comput Biol. 2011 Apr 28;7(4):e1002037. doi: 10.1371/journal.pcbi.1002037 (PMC3084228; doi:10.1371/journal.pcbi.1002037)
Supplement: Table S3 — Comparison of Autodock Vina energies normalized of Nelfinavir, Saquinavir and Indinavir when bound to EGFR, ErbB2 and ErbB4. (DOC) [file pcbi.1002037.s007.doc]

**Table S3.** Comparison of Autodock Vina energies normalized of Nelfinavir, Saquinavir and Indinavir when bound to EGFR, ErbB2 and ErbB4.

|  | Autodock Vina energy (kcal/mol) | | |
| --- | --- | --- | --- |
|  | EGFR | ErbB2 | ErbB4 |
| Nelfinavir | -9.4 | -9.0 | -9.1 |
| Saquinavir | -8.6 | -9.6 | -10.4 |
| Indinavir | -8.9 | -8.9 | -9.7 |
